# Development and Characterization of Novel Hybrid Particleboard Made from Several Non-Wood Lignocellulosic Materials

**DOI:** 10.3390/polym17040512

**Published:** 2025-02-16

**Authors:** Fazilla Oktaviani Tarigan, Luthfi Hakim, Agus Purwoko, Tito Sucipto, Halimatuddahliana Nasution, Widya Fatriasari, Muhammad Adly Rahandi Lubis, Jajang Sutiawan, Mohammad Irfan Bakhsi, Nam-Hun Kim, Petar Antov, Seng Hua Lee, Rangabhashiyam Selvasembian, Mohd Hazwan Hussin, Manggar Arum Aristri, Apri Heri Iswanto

**Affiliations:** 1 Department of Forest Products, Faculty of Forestry, Universitas Sumatera Utara, Kampus 2 USU Kuala Bekala, Medan 20355, Indonesia; fazillaoktaviani06@gmail.com (F.O.T.); luthfi@usu.ac.id (L.H.); aguspurwoko@usu.ac.id (A.P.); tito@usu.ac.id (T.S.); 2 Faculty of Engineering, Universitas Sumatera Utara, Kampus USU Padang Bulan, Medan 20355, Indonesia; halimatuddahliana@usu.ac.id; 3 Research Center for Biomass and Bioproducts, National Research and Innovation Agency, Cibinong 16911, Indonesia; widy003@brin.go.id (W.F.); muha142@brin.go.id (M.A.R.L.); jaja007@brin.go.id (J.S.); itzirfanchemscholar@gmail.com (M.I.B.); 4 Department of Forest Biomaterials Engineering, College of Forest and Environmental Sciences, Kangwon National University, Chuncheon 24341, Republic of Korea; kimnh@kangwon.ac.kr; 5 Faculty of Forest Industry, University of Forestry, 1797 Sofia, Bulgaria; p.antov@ltu.bg; 6 Department of Wood Industry, Faculty of Applied Sciences, Universiti Teknologi MARA (UiTM), Cawangan Pahang Kampus Jengka, Shah Alam 26400, Malaysia; leesenghua@hotmail.com; 7 Department of Environmental Science and Engineering, School of Engineering and Sciences, SRM University-AP, Amaravati 522240, India; rambhashiyam@gmail.com; 8 Materials Technology Research Group (MaTReC), School of Chemical Sciences, University Sains Malaysia, Minden, Gelugor 11800, Malaysia; mhh@usm.my; 9 Department of Forest Management, Faculty of Agriculture, Universitas Sebelas Maret, Ir Sutami 36A, Surakarta 57126, Indonesia; arumaristri@gmail.com

**Keywords:** acoustic properties, chemical properties, composite, hybrid particleboard, non-wood lignocellulosic, mechanical properties, physical properties

## Abstract

The green transition trend in the wood-based panel industry aims to reduce environmental impact and waste production, and it is a viable approach to meet the increasing global demand for wood and wood-based materials as roundwood availability decreases, necessitating the development of composite products as alternatives to non-wood lignocellulosic raw materials. As a result, the purpose of this study is to examine and assess the physical, mechanical, and acoustic properties of particleboard manufactured from non-wood lignocellulosic biomass. The core layer was composed of non-wood lignocelluloses (banana stem, rice straw, coconut fiber, sugarcane bagasse, and fibrous vascular bundles (FVB) from snakefruit fronds), whereas the surface was made of belangke bamboo (*Gigantochloa pruriens*) and wood. The chemical characteristics, fiber dimensions and derivatives, and contact angles of non-wood lignocellulosic materials were investigated. The contact angle, which ranged from 44.57 to 62.37 degrees, was measured to determine the wettability of these materials toward adhesives. Hybrid particleboard (HPb) or sandwich particleboard (SPb) samples of 25 cm × 25 cm with a target density of 0.75 g/cm^3^ and a thickness of 1 cm were manufactured using 7% isocyanate adhesive (based on raw material oven dry weight). The physical parameters of the particleboard, including density, water content, water absorption (WA), and thickness swelling (TS), ranged from 0.47 to 0.79 g/cm^3^, 6.57 to 13.78%, 16.46 to 103.51%, and 3.38 to 39.91%, respectively. Furthermore, the mechanical properties of the particleboard, including the modulus of elasticity (MOE), bending strength (MOR), and internal bond strength (IB), varied from 0.39 to 7.34 GPa, 6.52 to 87.79 MPa, and 0.03 to 0.69 MPa, respectively. On the basis of these findings, the use of non-wood lignocellulosic raw materials represents a viable alternative for the production of high-performance particleboard.

## 1. Introduction

Roundwood is becoming increasingly scarce due to high demand and limited supply. According to the Ministry of Environment and Forestry (2021), Indonesia produced 52.92 million m^3^ of roundwood in 2020, while the demand amounted to 60.3 million m^3^. This circumstance influences the wood processing industry significantly, as the high demand for roundwood, coupled with a restricted supply of raw materials, leads to reduced product output and increased prices [1]. According to Pędzik et al. [2], continued reliance on roundwood may limit future wood-processing production, highlighting the need for composite products as alternatives. Wood-based composites are materials created by combining wood fibers, particles, veneers, strands, or other types of lignocellulosic materials with synthetic and/or biobased adhesives, along with other components, to form a unified material. Composite materials can be utilized as alternatives to traditional wood products to address the sustainability and cost-effectiveness issues of the wood-processing industry; additionally, composites have greater durability. The manufacture of composite materials involves a variety of raw materials, one of which is non-wood lignocellulosic materials [3]. Tye et al. [4] reported that non-wood lignocellulosic materials have great potential as alternatives to roundwood and will play an important role in developing composite products such as particleboard. This is due to their greater availability and shorter growth cycles compared to trees. These materials include banana stems, rice straw, coconut husks, bagasse, and fibrovascular bundles (FVB) from snakefruit palm fronds. Farmers have not fully maximized the use of non-wood lignocellulosic materials obtained from agricultural commodities. However, lignocellulosic materials can be exploited to increase their economic value by producing particleboard.

Previous research by Iswanto et al. [5] on particleboard manufactured from non-wood lignocellulosic materials (rice husks) revealed low strength and dimensional stability, failing to meet the JIS A 5908-2003 criteria [6]. Silva et al. [7] reported that particleboard manufactured from bagasse had a density ranging from 0.34 to 0.44 g/cm^3^, whereas Kariuki et al. [8] reported lower mechanical characteristics. As a result, this study focuses on improving the physical and mechanical properties of particleboard derived from nonwood lignocellulosic materials by modifying it into sandwich particleboard (SPb). SPb is similar to comply boards but differs in its use of veneer and plywood. It consists of surface layers, a core, and a back joined together with an adhesive. Several studies have reported that SPb can improve the physical and mechanical properties of particleboard, particularly the board density. Furthermore, using strong materials for the surface layers of the boards demonstrates their ability to withstand loads [5,7,9,10,11,12,13,14].

Therefore, there is a need for technically feasible research on non-wood lignocellulosic materials such as banana stems, rice straw, coconut husks, bagasse, and snakefruit palm fronds as the core, with Belangke bamboo, meranti wood strands, and meranti veneers as surface layers. Bamboo and meranti wood were chosen as surface materials because of their high availability in North Sumatra. According to Iswanto and Anjarani [10], bamboo has notable strength advantages. However, Manik et al. identified it as a lightweight hardwood in strong class II wood, making it suitable for use as a laminated board [15]. This combination is predicted to enhance the dimensional stability of composite boards, meeting the requirements of the JIS A 5908-2003 standard [6].

## 2. Materials and Methods

### 2.1. Materials

**Figure 1:**
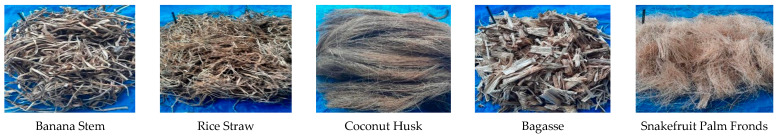
Non-wood lignocellulosic materials.

**Figure 2:**
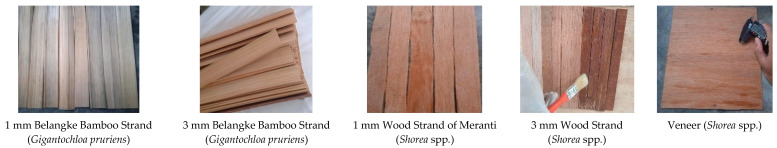
Raw materials used for the surface layers of the hybrid particleboard produced in this work.

In this research, the raw materials used for analysis included non-wood lignocellulosic materials for the core of the sandwich particleboard (Figure 1) and different raw materials for the surface layers of the particleboard (Figure 2). The characteristics of the nonwood lignocellulosic and surface materials are presented in Table 1 and Table 2. The dimensions (length, width, and thickness) of the non-wood lignocellulosic materials and the materials used for the surface layers were evaluated via digital calipers (Krisbow, Indonesia). Commercial isocyanate adhesive from PT. Polychemie Asia Pacific Permai (Jakarta, Indonesia) was utilized in this study. The isocyanate adhesive had a solids content of 98%, a viscosity of 150–250 cps at 23 °C, a pH of 6.5–8.5, a pot life mixture of ±60 min, a spread volume of 200–250 g/m^2^, an assembly time of ±10 min, a cold press of 0.69–1.47 MPa, and a cold press time of 30–45 min. In addition, the materials used for testing were solutions consisting of distilled water, hot water, cold water, 1:2 benzene ethanol, 17 and 8.3% NaOH, 10% acetic acid, H_2_SO_4_, 25% NaClO_2_, acetone, glacial acetic acid, hydrogen peroxide, alcohol, glycerin, and safranin.

### 2.2. Characterization of Raw Materials

#### 2.2.1. Sample Preparation and Chemical Characterization

The chemical components of lignocellulosic materials, including banana stems, rice straw, coconut fiber, sugarcane pulp, and FVB snakefruit stems, were evaluated. Sample preparation was based on the Technical Association of the Pulp and Paper Industry (TAPPI) T257 cm-02 and TAPPI T 264 cm-97 standards [21,22]. During sample preparation, the lignocellulosic materials used for testing the chemical properties were considered representative of the whole material. Five types of samples were used: bagasse, rice straw, coconut fiber, banana stems, and snakefruit fronds. The samples were ground via a ring flaker/hammer with particle sizes ranging from 40 to 60 mesh. These samples were then used for further testing, including analyses of lignin, acid-soluble lignin, holocellulose, alpha-cellulose, extraction, and ash content. The lignin content was determined according to the National Renewable Energy Laboratory (NREL) Laboratory Analytical Procedure (LAP) 003 standard [23], the holocellulose content was tested according to Paper Trade J by Wise et al., 1946 [24], the alpha-cellulose content was determined at the Chemical Rubber Company (CRC) Press by Rowell in 2005 [25], and the extractive content in ethanol–benzene with a ratio of 1:2 was determined on the basis of the TAPPI 204 cm-97 standard [26]. The ash content was tested according to the TAPPI T 211 cm-02 standard [27], with samples dried in a furnace at 525 °C for 30 min and then cooled in a desiccator for 60 min. All the measurements were repeated three times for each sample, and the results of the chemical composition analyses were expressed relative to the oven-dry weight of the wood.

#### 2.2.2. Degree of Crystallinity and Fiber Dimensions

The degree of crystallinity was estimated from the intensity data from X-ray diffraction (XRD) analysis. In this work, XRD analysis of the compound composition was conducted on samples after drying. An electrical current of 40 kV and 30 mA were used. XRD (MaximaX-XRD 700, Shimadzu, Japan) was performed with a CuK X-ray source (0.15406 nm). Initially, 5 g of sample with a particle size of 40 mesh was placed in the sample holder and evaluated at room temperature with a scanning speed of 2°/min and a scanning angle of 10–40°.

The fiber dimensions were observed via maceration preparations according to the Schultze method. Each sample of lignocellulosic material was cut into 5 cm pieces and gradually heated in a reaction tube containing a mixture of hydrogen peroxide solution and glacial acetic acid at a 1:1 ratio (*v*/*v*). The fibers were washed with flowing water and stained with safranin. The sample slides were treated with glycerin, after which the stained fibers were loaded and covered with a cover slip. A total of 50 individual fibers from each segment were used to evaluate the fiber dimensions, including the fiber length and diameter, and were subsequently observed under a ZEISS Primostar microscope (ZEISS AG, Oberkochen, Germany) [28]. Other parameters, such as the Runkel ratio, the felting power/slenderness ratio, the Muhlsteph ratio, the coefficient of rigidity, and the flexibility ratio, include the lumen diameter and cell wall thickness, which are used to obtain derivative values. The calculated fiber dimension derivative values were the Runkel ratio (2 × cell wall thickness/lumen diameter), the felting power (fiber length/fiber diameter), the Muhlsteph ratio {(fiber diameter squared-lumen diameter squared)/(fiber diameter squared)} × 100%, the coefficient of rigidity (fiber wall thickness/fiber diameter) and the flexibility ratio (lumen diameter/fiber diameter) [29].

#### 2.2.3. Contact Angle

**Figure 3:**
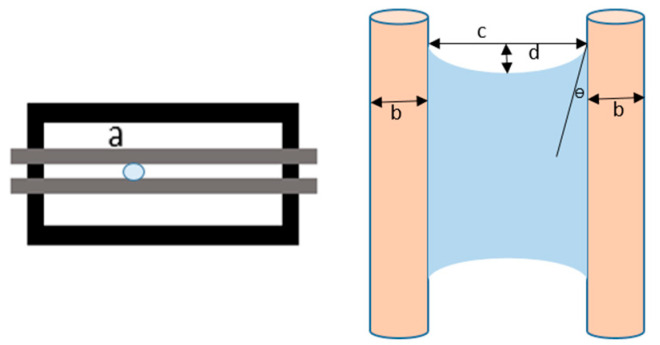
Calculation of contact angle. Description: a: liquid (adhesive), b: fiber diameter, c: distance between fibers, d: contact angle of water with fiber, e: depression from the meniscus formed by water.

For contact angle measurements via the Schellbach et al. [30] method, lignocellulosic materials with a size of approximately 5 cm were prepared, with 3 replications. Two fibers with the same diameter were subsequently arranged parallelly and glued to the sample frame under a microscope, separated by a distance of 0.2–1.0 mm. Isocyanate adhesive was dropped between the two fibers to obtain a liquid hanging on both fibers, which was then photographed via an SLR digital camera Nikon D5600 (Nikon, Nishioi, Shinagawa-ku, Tokyo, Japan). For image analysis, IC-Measure software version 2.0.0.245 (Imaging Source, Bremen, Germany) was used to measure the fiber contact angle. Measurements were taken at 4 angles between the fibers and the contact liquid (Figure 3). The results of the contact angle calculations between the non-wood lignocellulosic materials and the isocyanate adhesive are presented in Table 3. Table 3 shows the contact angle values of the four tested samples. The average values of the four angles and three repetitions tested on each sample are summarized.

### 2.3. Production of Sandwich Particleboard (SPb)

**Figure 4:**
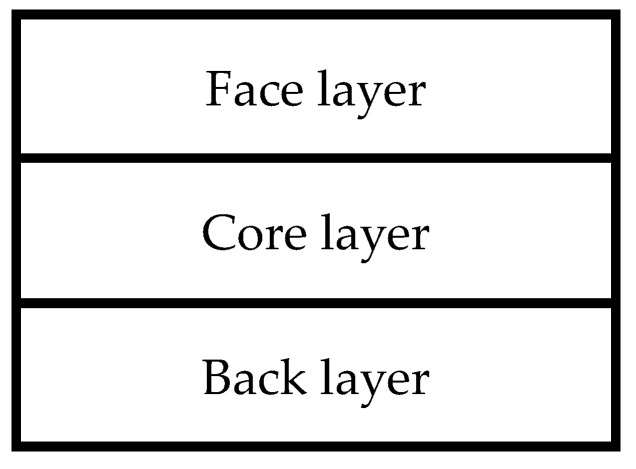
Three-layer particleboard.

SPb panels were manufactured with dimensions of 25 cm × 25 cm, a target thickness of 1 cm, and a target density of 0.75 g/cm^3^. These panels were made in three layers using 7% isocyanate adhesive (solids content 98%) (Figure 4). The particle requirements were calculated by including the components of the board area (25 cm × 25 cm), target density (0.75 g/cm^3^), and board thickness (1 cm). Moreover, the adhesive requirement was calculated on the basis of the dry weight of the particles and the adhesive content used. The raw material and adhesive quantities used for manufacturing the SPb panels are shown in Table 4.

The surface layers (face and back) were made of bamboo strands, wood, and veneer, whereas the core layer comprised banana stems, rice straw, bagasse, coconut husks, and snakefruit palm fronds.

The particles and adhesive were mixed evenly via a spray gun machine (Meiji, Japan) and applied over one large area for the strands or veneers used as surface layers. The sheets were subsequently made by placing the mixed particles into 25 × 25 cm^2^ molds. After molding, the boards were compressed via a hot press at a temperature of 160 °C for 5 min at a pressure of 2.94 MPa. Four replications of boards were made, comprising three physical and mechanical tests and one acoustic property test. Therefore, the total number of boards produced in this research was 120, considering 5 factors (L) × 6 factors (P) × 4 replications. The finished boards were conditioned for 14 days at a room temperature of approximately 26 ± 2 °C and a relative humidity of approximately 60%. This process was carried out to standardize the moisture content and eliminate residual stresses formed during hot pressing.

### 2.4. Characterization of Sandwich Particleboard

#### 2.4.1. Physical and Mechanical Properties

After being subjected to a conditioning process for 14 days, the boards were cut into test samples of various sizes according to the JIS A 5908 (2003) standard [6]. The physical properties tested included density, moisture content, water absorption, and thickness swelling. The mechanical properties tested were the modulus of elasticity (MOE), modulus of rupture (MOR), and internal bond strength (IB). The physical and mechanical properties of the SPb were subsequently tested according to the JIS 5908-2003 standards [6] (Table 5).

The density of the board was compared in terms of mass and volume. Following a 24-h drying period at 103 ± 2 °C, the original and final masses were used to compute the moisture content. The springback was determined by measuring the differences in board thickness before and after conditioning [24]. The difference between the masses before and after soaking for 24 h was used to calculate the water absorption values. Thickness swelling was determined by measuring the difference between the initial thickness before soaking and after 24 h. A universal testing machine RTH-1350 (A & D Company, Tokyo, Japan) with a 10 mm/min loading speed was used to perform the MOE and MOR tests. The IB test was conducted via a universal testing machine with a 2 mm/min loading speed.

#### 2.4.2. Sound Absorption Properties

The samples used for the acoustic tests were cut into circular patterns with diameters of 3 cm and 10 cm. The test was carried out using 3 cm samples for the 250–5250 Hz range, whereas those with a diameter of 10 cm were applied for frequencies of 100–1600 Hz, according to BS EN ISO 10,534 (2001) [31]. An impedance tube was subsequently used to measure the acoustic parameters of small samples according to the tube size and with the sound direction perpendicular to the surface. The absorption coefficient was calculated by measuring the sound pressure incident on the material surface and reflected by the test materials.

### 2.5. Data Analysis

#### 2.5.1. Test of Chemical Properties

The chemical properties of the nonwood lignocellulosic materials were further subjected to a *t*-test to determine significant differences between the types of raw materials. The data obtained were processed and analyzed via computerized software such as SPSS Version 23. The decision-making basis for the independent samples *t*-test is as follows:

a. When the Sig value (2-tailed) < 0.05, H0 is rejected.

b. When the Sig value (2-tailed) > 0.05, H0 is accepted.

#### 2.5.2. Completely Random Design of Sandwich Particleboard

The research method used a complete factorial random design with two treatment factors, namely, the type of nonwood lignocellulosic material and the surface layer type for SPb.

## 3. Results and Discussion

### 3.1. Alpha-Cellulose

**Figure 5:**
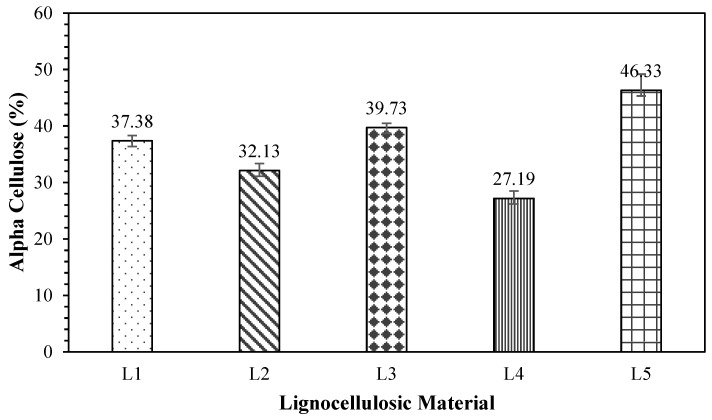
Alpha-cellulose content in non-wood lignocellulosic materials. L1: banana stem; L2: rice straw; L3: coconut husk; L4: bagasse; L5: snakefruit palm frond FVB.

Alpha-cellulose is a component that serves a structural function in nonwood lignocellulosic materials, with the highest value among other components. The contents of the tested non-wood lignocellulosic materials are presented in Figure 5. The alpha-cellulose content of the tested nonwood lignocellulosic materials ranged from 27.19% to 46.33%. Snakefruit palm fronds had the highest content of 46.33%, while the lowest was found in bagasse, at 27.19%. Etale et al. [32] reported that cellulose is a hydrophilic polymer with reactive hydroxyl groups in each hydroglucose unit, leading to the absorption of water or certain solvents and swelling of the cellulose polymer. Therefore, a higher alpha-cellulose content in materials, including non-wood lignocellulosic materials, contributes to increased quality in terms of strength [33]. Table 6 shows the *t*-test results for the alpha-cellulose content in non-wood lignocellulosic materials. The statistical results in Table 6 revealed that the alpha-cellulose content of bagasse was not significantly different from that of rice straw but varied from that of coconut husks, banana stems, and snakefruit palm fronds.

### 3.2. Hemicelluloses

**Figure 6:**
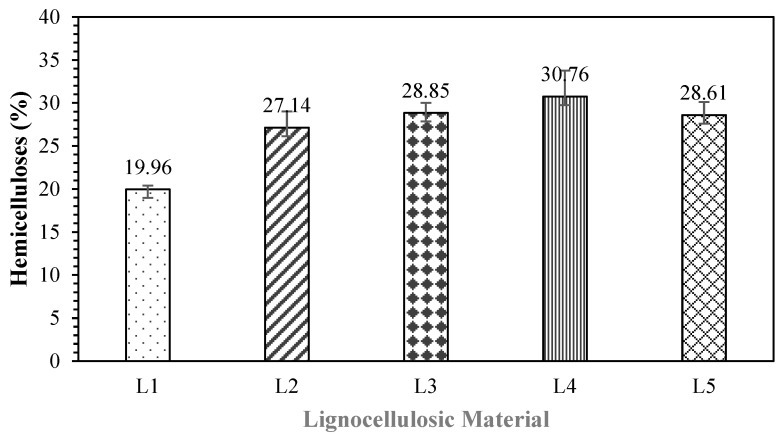
Hemicellulose content in non-wood lignocellulosic materials. L1: banana stem; L2: rice straw; L3: coconut husk; L4: bagasse; L5: snakefruit palm frond FVB.

Non-wood lignocellulosic materials include polysaccharides, a combination of monosaccharide monomers that form polymers containing cellulose and hemicellulose. Specifically, hemicelluloses are heteropolysaccharides and important components of nonwood lignocellulosic materials. Hemicelluloses are isolated from holocellulose by sequential extraction with dimethyl sulfoxide and an alkali solution that can be used for precipitation. The hemicellulose contents of the tested non-wood lignocellulosic materials are presented in Figure 6.

The hemicellulose content of the raw materials ranged from 19.96% to 30.76%, with the highest values found in sugarcane bagasse and the lowest in banana stems. Moreover, the hemicellulose content differs between holocellulose and alpha-cellulose. Based on the research conducted by Lamaming et al. [34], during the heating process, the chain scission that occurs in hemicelluloses does not reduce the strength of particles compared to alpha-cellulose. Therefore, hemicelluloses are essential in the pressing process of particleboard made without adhesive. Widyorini et al. [35] reported that the degradation of hemicelluloses in the pressing stage can produce furans (heterocyclic organic compounds), which play a role in bonding in the particleboard manufacturing process. Table 7 shows the results of the *t*-test used to determine the statistically significant differences in hemicellulose content; bagasse was found to differ substantially from banana stems but not from coconut husks, rice straw, or snakefruit palm fronds.

### 3.3. Holocellulose

**Figure 7:**
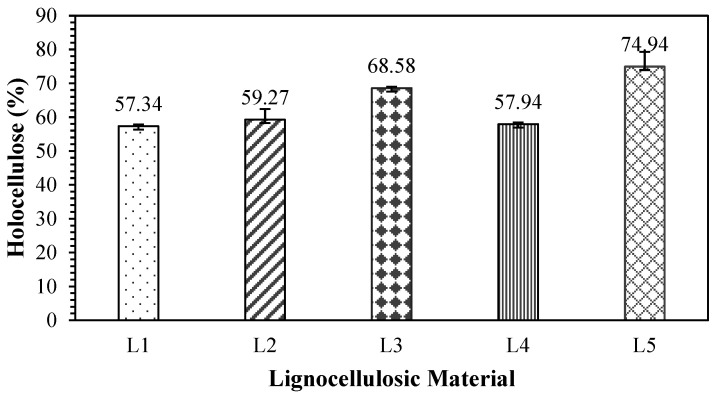
Holocellulose content in non-wood lignocellulosic materials. L1: banana stem; L2: rice straw; L3: coconut husk; L4: bagasse; L5: snakefruit palm frond FVB.

Holocellulose is the total polysaccharide content of alpha-cellulose and hemicelluloses contained in materials. The results of the determination of the holocellulose content of the lignocellulosic materials tested are shown in Figure 7 and Table 8. The holocellulose content in the tested non-wood lignocellulosic materials ranged from 57.34% to 74.94%, with the highest value in snakefruit palm fronds and the lowest in banana stems. This research revealed a linear pattern in the holocellulose content, with the highest alpha-cellulose content occurring in the raw materials, particularly in the snakefruit palm fronds. According to Mohd et al. [36], holocellulose consists of cellulose and hemicelluloses, which are linearly correlated. Angelini et al. [37] reported that holocellulose is closely related to the lignin content in lignocellulosic materials, suggesting a low lignin content.

### 3.4. Lignin

**Figure 8:**
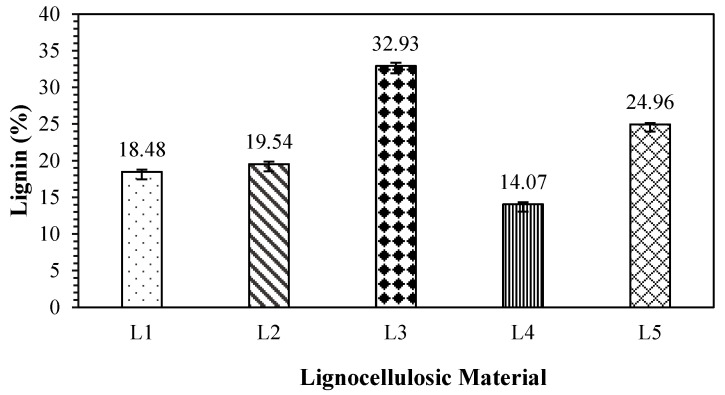
Lignin content in non-wood lignocellulosic materials. L1: banana stem; L2: rice straw; L3: coconut husk; L4: bagasse; L5: snakefruit palm frond FVB.

Lignin is found in the cell walls of lignocellulosic materials and is a binder. Furthermore, it plays a role in determining the properties of materials, as a low lignin content is soft. The values of lignin content obtained in this research are shown in Figure 8. Figure 8 shows that the lignin content of the tested non-wood lignocellulosic materials ranged from 14.07% to 32.93%, with the highest lignin content found in coconut husks and the lowest in bagasse. According to Çetin and Özmen [38], lignin has an aromatic structure and a high degree of cross-linking, similar to phenol-formaldehyde (PF) resin. Previous studies have used lignin as a substitute for resin because of its effect on particle bonding in particleboard production. Similarly, Birch et al. [39] reported that lignin provided resistance to compressive forces in material structures. This was also supported by Gindl et al. [40], where lignin was found to contribute significantly to the rigidity of cell walls. Table 9 shows the *t*-test results for the lignin content of non-wood lignocellulosic materials. The lignin content of bagasse significantly differed from that of coconut husk, banana stem, rice straw, and snakefruit palm fronds.

### 3.5. Extractives

**Figure 9:**
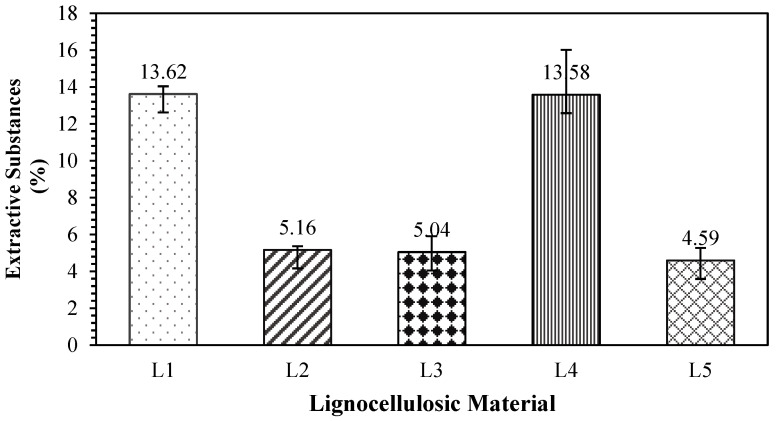
Extractive substance contents in non-wood lignocellulosic materials. L1: banana stem; L2: rice straw; L3: coconut husk; L4: bagasse; L5: snakefruit palm frond FVB.

The values of the extracted substances contained in the lignocellulosic materials are presented in Figure 9. The content of extractive substances in the non-wood lignocellulosic materials ranged from 4.59% to 13.62%, with the highest value in banana stems and the lowest in snakefruit palm fronds. Tye et al. [4] reported that the higher content of extractive substances in non-wood lignocellulosic materials contributed to the inhibition of adhesive bonding between particles during particleboard manufacturing. The extractive substance content in non-wood lignocellulosic materials affects water absorption, as low values can reduce the equilibrium moisture content contained in boards [41]. Table 10 shows that the *t*-test results for the extractive substance content of bagasse are not significantly different from those of banana stems but vary from those of coconut husks, rice straw, and snakefruit palm fronds.

### 3.6. Ash Content

**Figure 10:**
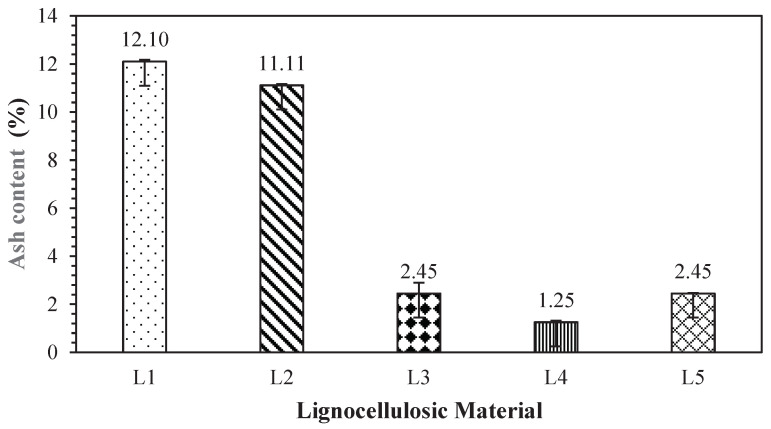
Ash content of non-wood lignocellulosic materials. L1: banana stem; L2: rice straw; L3: coconut husk; L4: bagasse; L5: snakefruit palm frond FVB.

The ash content represents the inorganic residue remaining after the combustion of organic materials. The values of ash content obtained in this research are presented in Figure 10. The ash content of non-wood lignocellulosic materials ranged from 1.25 to 12.10%, with the highest ash content in banana stems and the lowest in bagasse. According to Kurokochi and Sato [42], the ash content of non-wood lignocellulosic materials is related to the internal bond of the particleboard, with higher ash content reducing the internal bond values. Similarly, Segovia et al. [43] reported that a high ash content reduces adhesion reactivity to polar adhesives, affecting the internal bond strength of particleboard. The *t*-test results shown in Table 11 indicate that the ash content of bagasse is not significantly different from that of coconut husks but varies from that of banana stems, rice straw, and snakefruit palm fronds.

### 3.7. Degree of Crystallinity

The degree of crystallinity is calculated by determining the ratio of the crystalline fraction area to the sum of both the crystalline and amorphous fraction areas. The determined crystallinity values of the non-wood lignocellulosic materials tested are shown in Table 12. Table 12 shows that the crystallinity values range from 21.13% to 35.34%, with the highest value obtained in snakefruit palm fronds and the lowest in rice straw. The crystalline region is an area with a regular and long-range arrangement of atoms. Research has shown that the presence of more crystalline regions in materials significantly influences the superior physical properties of polymers [44]. Moreover, the degree of crystallinity is also related to the moisture content of non-wood lignocellulosic materials. During heating, the degree of crystallinity tends to decrease due to the reduction in the crystalline diameter. Akpan et al. [45] reported that amorphous lignin melts during heating and moves to the surface, covering the fibers and leading to surface changes. This observation is consistent with the decrease in the moisture content of non-wood lignocellulosic raw materials after heat treatment, which disrupts the hydrogen bonds between molecules in the hemicelluloses/lignin matrix.

### 3.8. Lignocellulosic Fiber Dimensions

**Figure 11:**
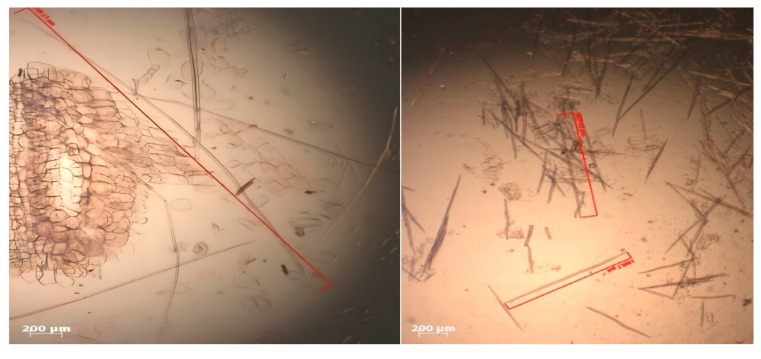
Fiber images of banana stems and coconut husks.

The quality of a fiber can be determined by its length, diameter, lumen, and wall thickness in micrometers (μm) (Table 13). These parameters and their derivatives, including tensile strength, the Muhlstep ratio, flexibility, the Runkel number, and the coefficient of rigidity, are presented in Table 14. According to Table 13, the length of non-wood lignocellulosic fibers ranged from 1020.36 to 4033.79 μm. On the basis of the criteria and quality of wood fiber classification, all non-wood lignocellulosic materials tested in this research can be classified into class I, with fiber lengths >2000 μm, except for coconut husk, which is classified into class II [46]. Supartini et al. [46] reported that fiber length could affect physical properties such as strength and rigidity, with longer fibers showing broader bonding and better pressure distribution properties. Figure 11 shows that the longest and shortest fibers among the non-wood lignocellulosic materials tested were banana stems at 4033.79 μm and coconut husks at 1020.36 μm. The fiber length can influence the flexural strength of particleboard. In this research, the fiber diameter ranged from 16.92 to 64.98 μm, with the smallest value found in rice straw and the largest in sugarcane. The fiber diameter affects the derived values, as a larger diameter results in higher Muhl step ratios. Moreover, smaller values lead to higher tensile strengths, flexibility ratios, and coefficients of rigidity [47].

**Figure 12:**
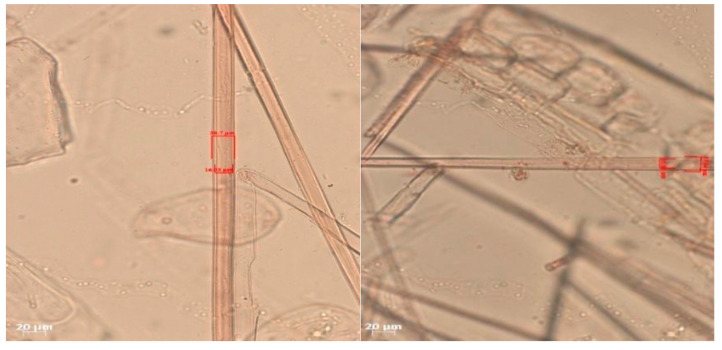
Banana stem and rice straw lumens.

The lumen diameter of non-wood lignocellulosic materials ranged from 10.98 to 16.34 μm, with the largest diameter found in rice straw at 16.34 μm and the smallest diameter in banana stems at 10.98 μm, as shown in Figure 12. Moreover, the lumen diameter values also influence the derived values of the fiber dimensions. The thickness of the fiber walls from the nonwood lignocellulosic materials ranged from 2.97–4.71 μm, with the thickest being found in sugarcane and the thinnest being observed in rice straw. Sandri and Maideliza [48] reported that thicker fiber walls and thinner lumens resulted in materials with high density and hardness. On the basis of the data in Table 14, the values derived for the fiber dimensions of nonwood lignocellulosic materials revealed that the Runkel number ranged from 0.41 to 0.82. This Runkel value is obtained from the ratio of twice the fiber wall thickness to the lumen diameter. Consequently, a high Runkel value indicates thicker cell walls, whereas a smaller lumen diameter reduces the Runkel number.

The smelting power values obtained ranged from 46.79 to 181.1, with the highest value found in the banana stems and the lowest in the coconut husks. According to Supartini et al. [46], felting power determines flexibility properties, and good weaving can be obtained from longer fibers. The Muhlstep ratio ranged from 48.61 to 89.67, with the lowest value found in the banana stem and the highest in the snakefruit palm frond. Aprianis et al. [49] reported that a smaller Muhlstep ratio resulted in better strength properties. A comparison of the flexibility properties was used to compare the differences between the lumen and fiber diameters. The flexibility values of non-wood lignocellulosic materials ranged from 0.32 to 0.71. A high flexibility value corresponds to a thin wall thickness and easy deformability, which are typically associated with good tensile strength [50]. The coefficient of rigidity of the non-wood lignocellulosic materials ranged from 0.16 to 0.22, with the lowest value found in banana stems and the highest in bamboo. This coefficient is obtained from the comparison of the cell wall thickness with the fiber diameter, which is negatively correlated with the tensile strength. The properties of fibers during production significantly influence the overall quality of particleboard, affecting its mechanical strength, dimensional stability, and surface quality. The quality of particleboard fundamentally depends on the properties of the fibers used in its composition. Optimizing the fiber size, type, distribution, density, and moisture content can significantly improve the mechanical strength, dimensional stability, and surface quality of particleboard [42,43].

### 3.9. Contact Angle

**Figure 13:**
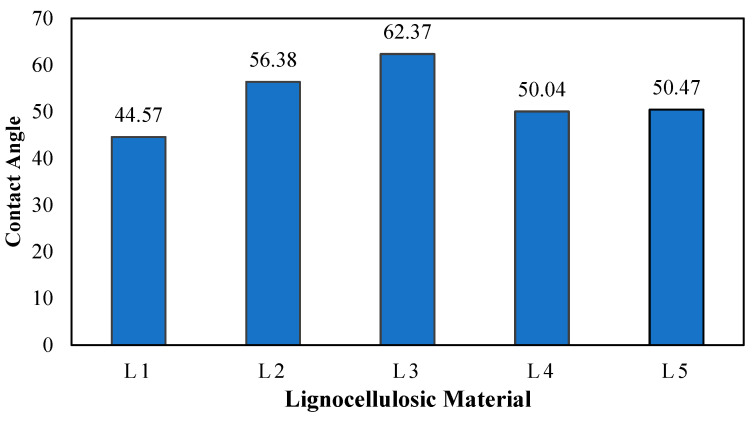
Contact angle values for the non-wood lignocellulosic materials: L1: banana stem, L2: rice straw, L3: coconut husk, L4: bagasse, L5: snakefruit palm frond FVB.

The contact angle is formed by the liquid phase between the surface of a solid object and the tangent line from the liquid droplet radius, which touches the contact point of a solid [51]. Figure 13 shows the contact angle values of the non-wood lignocellulosic materials tested, which ranged from 44.57° to 62.37°, with the highest value obtained in the coconut husks and the lowest in the banana stems. Generally, the contact angle is measured to determine the wettability of non-wood lignocellulosic materials toward adhesives. Research has shown that a smaller contact angle results in better wettability [52]. Syamani et al. [53] reported that higher contact angles between adhesive solutions and fibers could increase fiber wettability. This increase is attributed to the loss of wax and pectic substances on the surface for smoothness to enhance the wettability of the adhesive. According to Tang et al. [54], the contact angle is closely related to adhesive penetration on the surface, which significantly affects the adhesion strength.

### 3.10. Physical Properties of the Particleboard

#### 3.10.1. Density

**Figure 14:**
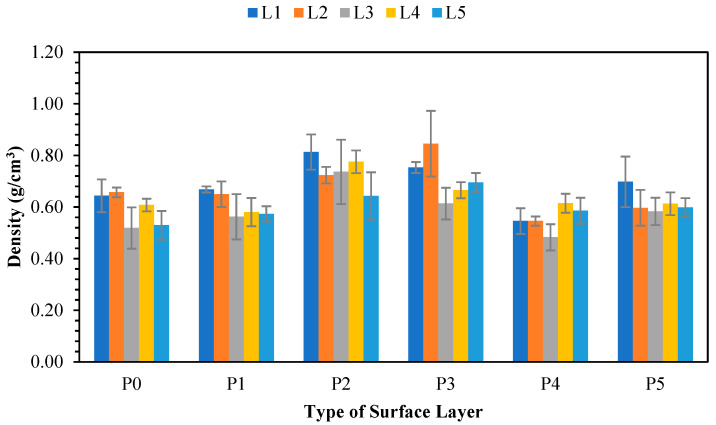
Density of SPb fabricated from non-wood lignocellulosic materials and various types of surface layers. P0: without surface layers, P1: 1 mm bamboo strand, P2: 3 mm bamboo strand, P3: 1 mm wood strand, P4: 3 mm wood strand, P5: veneer, L1: banana stem, L2: rice straw, L3: coconut husk, L4: bagasse, L5: snakefruit palm fronds FVB.

A graphical representation of the density values of the laboratory-made non-wood lignocellulosic particleboards manufactured with different types of surface layers (without adhesive, 1 mm bamboo, 3 mm bamboo, 1 mm wood strand, 3 mm wood strand, and veneer) is given in Figure 14. The density values of the tested samples met the JIS A 5908:2003 standard [6], which requires a range from 0.40 to 0.90 g/cm^3^, but did not achieve the target of 0.75 g/cm^3^. SPb, which exhibited a greater density than the target value, included banana stems and bagasse with 3 mm bamboo strand surface layers (0.81 and 0.78 g/cm^3^), as well as banana stems and rice straw with 1 mm wood strands at values of 0.75 and 0.85 g/cm^3^, respectively. Most of the particleboard produced did not meet the target board density of 0.75 g/cm^3^. This is thought to be due to the high springback value of the board. As shown in Table 15, almost all the boards made from coconut husk (L3) exhibited the highest springback values, which impacted the lower density value produced. The high springback value was attributed to the adhesive bond between the adhesive and the particles. The results indicated that coconut husk (L3) had the highest contact angle value among the materials tested. This suggests that its wettability is low, leading to weak interparticle bonds. Iswanto et al. [9] reported that springback occurred during compression and conditioning, causing the board thickness to increase from the target thickness after conditioning due to the effect of lignocelluloses. However, the initial condition of nonwood lignocellulosic materials is voluminous.

The analysis of variance results revealed that the treatment of non-wood lignocellulosic materials, the surface, and the interaction between the two treatments significantly affected the particleboard density, as presented in Table 16. Table 16 shows that non-wood lignocellulosic materials, the type of surface layers, and the interaction between the two treatments did not significantly affect the particleboard density. The highest density was obtained in the interaction treatment of L2P3 (rice straw and 1 mm wood) with L1P2 (banana stem and 3 mm bamboo), with values of 0.81 g/cm^3^ and 0.85 g/cm^3^, respectively. Moreover, the density of raw materials has a significant influence on particleboard, with low values resulting in a high compaction ratio [55]. In this study, the lowest particleboard density (0.48 g/cm^3^) was obtained in the L3P4 interaction treatment (coconut husk and 3 mm wood). Coconut husk has a relatively large volume per gram in the board manufacturing process [56]. Buranov et al. [57] explained the factors influencing the density of particleboard, including compaction pressure, particle amount, adhesive amount, and additives. Sitanggang et al. [58] reported an uneven adhesive distribution, leading to nonuniform pressure and heat during compaction, resulting in particleboard with the same volume but different weights.

#### 3.10.2. Moisture Content

**Figure 15:**
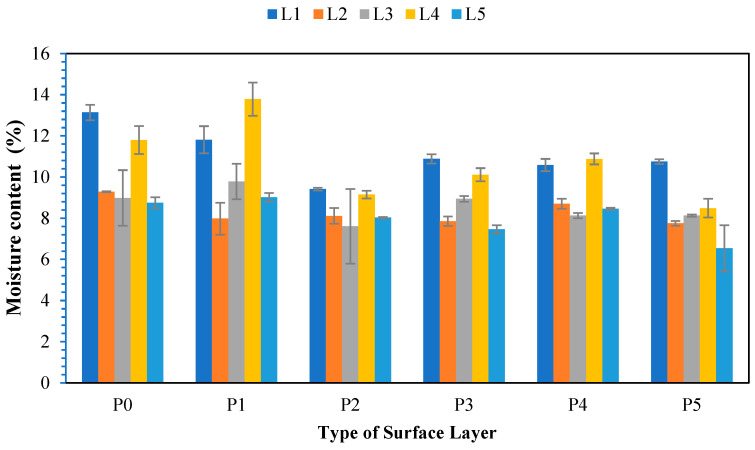
Moisture content of SPb fabricated from non-wood lignocellulosic materials and various types of surface layers. P0: without surface layers, P1: 1 mm bamboo strand, P2: 3 mm bamboo strand, P3: 1 mm wood strand, P4: 3 mm wood strand, P5: veneer, L1: banana stem, L2: rice straw, L3: coconut husk, L4: bagasse, L5: snakefruit palm fronds FVB.

Moisture content plays a significant role in material characteristics and derived properties. Acharjee et al. [59] reported that materials with lower moisture contents tend to have relatively more stable properties and experience reduced levels of biological degradation. The moisture content test results for the non-wood lignocellulosic materials and various surface layers are presented in Figure 15. The results shown in Figure 15 indicate that the moisture content of SPb ranged from 6.54% to 13.78%. These values almost fulfilled the JIS A 5908:2003 standard requirements, which specified a range of 5–13% for particleboard, except for banana stems without surface layers and bagasse of 1 mm bamboo. Mirza et al. [60] reported that raw materials can cause variations in the moisture content of particleboard. This is because the adhesive becomes more diluted when the raw materials have a high moisture content.

Table 17 shows that non-wood lignocellulosic materials, the type of surface layers, and their interactions did not significantly affect the moisture content of the SPb. The best moisture content of 6.54% was observed in L5P5 (Snakefruit palm fronds FVB particleboard with veneer surface layers). This finding is also consistent with the crystallinity values of snakefruit palm fronds FVB, as shown in Table 12, which are the highest of the other nonwood lignocellulosic materials. Therefore, when lignocellulosic materials are heated, the degree of crystallinity decreases, causing a reduction in crystallite diameter. In addition, the degree of crystallinity of materials is intricately linked to their chemical composition. Factors such as molecular symmetry, functional groups, chain configuration, and the presence of additives or impurities all play a role in determining how molecules pack together into ordered, crystalline regions. Understanding this relationship is crucial in tailoring material properties for specific applications, such as improving the strength of fibers, optimizing the thermal resistance of polymers, or enhancing the barrier properties of composites [61]. Generally, the moisture content of particleboard is lower than that of raw materials because of evaporation during the hot-pressing process. According to Khanchi and Birrell [62], the factors affecting the moisture content of non-wood lignocellulosic materials include the environment during drying.

#### 3.10.3. Water Absorption

**Figure 16:**
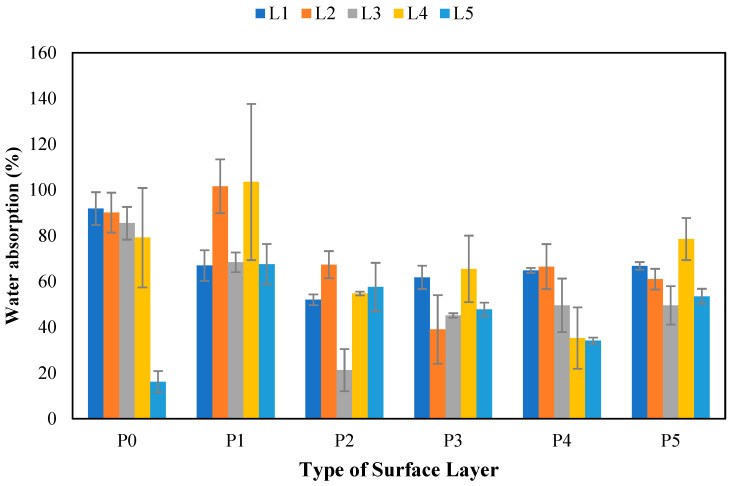
Water absorption of SPb fabricated from non-wood lignocellulosic materials and various types of surface layers. P0: without surface layers, P1: 1 mm bamboo strand, P2: 3 mm bamboo strand, P3: 1 mm wood strand, P4: 3 mm wood strand, P5: veneer, L1: banana stem, L2: rice straw, L3: coconut husk, L4: bagasse, L5: snakefruit palm fronds FVB.

The water absorption test results for the non-wood lignocellulosic materials and various surface layers are presented in Figure 16. Figure 16 shows that the water absorption values on the particleboard ranged from 16.46 to 103.51% [63]. Dina et al. [64] reported that the water absorption capacity of particleboard can be influenced by the particle size. Therefore, shorter particles tend to have a higher weight percentage, leading to a larger surface area for water absorption. This research revealed that the particleboard with the highest WA values was bagasse and banana stems with 1 mm bamboo strand layers. This occurred because the crystallinity percentage of bagasse and banana stems was lower than that of the non-wood lignocellulosic materials tested. Consequently, the amorphous regions in both materials are high and have the potential to absorb more water. Table 18 shows that non-wood lignocellulosic materials, surface layers, and the interaction between the two treatments significantly affect the water absorption of the particleboard. The results revealed that the highest water content of 103.51% was found in bagasse with a 1 mm bamboo strand surface layer. Wirawan et al. [65] reported that high water absorption was due to the hygroscopic nature of bagasse. Furthermore, the high value is attributed to the manual application of adhesive, which results in uneven distribution on surface layers, creating gaps for water absorption [66].

#### 3.10.4. Thickness Swelling

**Figure 17:**
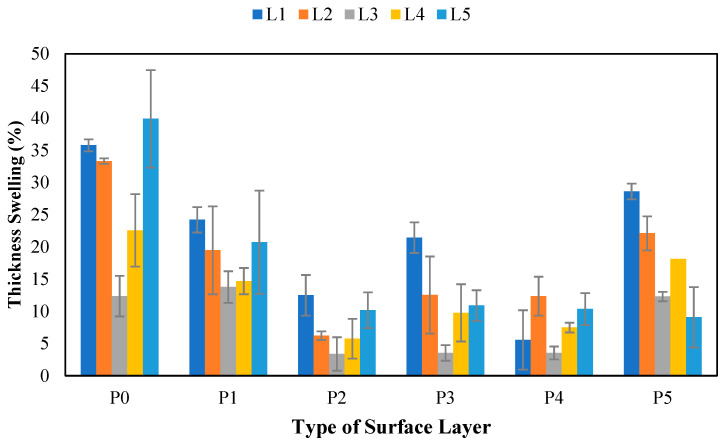
Thickness swelling of SPb fabricated from non-wood lignocellulosic materials and various types of surface layers. P0: without surface layers, P1: 1 mm bamboo strand, P2: 3 mm bamboo strand, P3: 1 mm wood strand, P4: 3 mm wood strand, P5: veneer, L1: banana stem, L2: rice straw, L3: coconut husk, L4: bagasse, L5: snakefruit palm fronds FVB.

Thickness swelling is attributed to water absorption, which causes the cell walls to expand, increasing the thickness swelling value of the board. The thickness swelling values of SPb obtained in this research are shown in Figure 17. The thickness swelling values of the tested SPs ranged from 3.38% to 39.91%. However, some boards, including banana stems without surface layers, did not meet the JIS A 5908:2003 standard [6], which requires thickness swelling not exceeding 12%. This finding shows that boards treated with surface layers can reduce the degree of thickness swelling. The application of surface layers to SPb plays a role in providing better water resistance. This phenomenon causes a reduction in swelling during soaking, leading to improved water resistance and reduced thickness. Additionally, the interaction and distribution of raw materials affect thickness swelling and dimensional stability [67]. Table 19 shows that non-wood lignocellulosic materials, surface layers, and the interaction between the two treatments did not significantly affect the thickness. The lowest swelling value was found in the L3P3 treatment (coconut husk with 3 mm bamboo surface layers), with a thickening development value of 3.38%. This was related to the higher lignin content in the coconut husks than in the other materials, as shown in Figure 8. Moreover, the natural hydrophobic properties of lignin indicate its water-repellency effectiveness [68].

The highest degree of board swelling was found in L1P0 and L5P0, with thicknesses of 35.78% and 39.91%, respectively. This high value was attributed to the higher fiber content in the banana stems than in the coconut husks, leading to dimensional swelling when the particleboard absorbs water. According to Garat et al. [69], water vapor absorption causes the fiber cell walls to swell, increasing thickness swelling. Figure 9 shows that banana stems have the highest extractive substance content, at 13.62%, among the other nonwood lignocellulosic materials. Extractive substances serve as an inhibiting factor in the bonding process, as lower values correlate with less broad swelling. Similarly, Akinyemi et al. [70] reported that non-wood lignocellulosic materials with high extractive substance contents should undergo pretreatment processes such as immersion in acetic acid and NaOH to produce boards with low thickness swelling. In addition, the particleboard density is a crucial factor influencing its thickness swelling behavior. Higher-density boards generally exhibit superior dimensional stability because of their reduced porosity, stronger bonding, and limited water absorption [71].

### 3.11. Mechanical Properties of the Particleboard

#### 3.11.1. Modulus of Elasticity (MOE)

**Figure 18:**
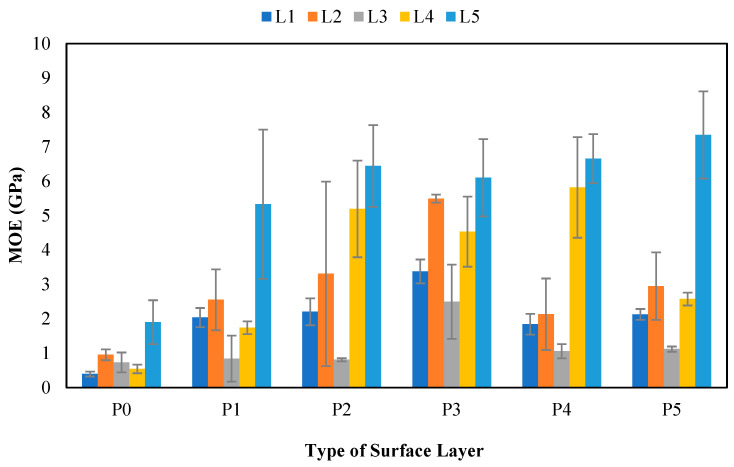
MOE of SPb fabricated from non-wood lignocellulosic materials and various surface layers. P0: without surface layers, P1: 1 mm bamboo strand, P2: 3 mm bamboo strand, P3: 1 mm wood strand, P4: 3 mm wood strand, P5: veneer, L1: banana stem, L2: rice straw, L3: coconut husk, L4: bagasse, L5: snakefruit palm fronds FVB.

Figure 18 shows that the MOE values ranged from 0.39 to 7.34 GPa. On the basis of these results, the MOE values of SPb without surface layers did not meet the JIS A 5908:2003 standard [6] of values greater than 2000 N/mm^2^ (2 GPa). In contrast, SPb with surface layers almost entirely met the standards. This shows that applying surface layers to SPb significantly affects the MOE values. According to the basic principle of mechanical properties, the MOE value is generated from the surface layers of the board that directly interact with the load received [10]. Table 20 shows that lignocellulosic materials, surface layers, and interactions between the two treatments do not significantly affect the MOE of SPb. The highest MOE value of 7.34 GPa was found in L5P5 (snakefruit palm frond FVB with 1 mm veneer surface layers). The MOE value of the particleboard with the FVB snakefruit palm frond mixture was the highest due to the high alpha-cellulose content. Generally, alpha-cellulose and lignin are the framework components of non-wood lignocellulosic materials, as higher values correlate with improved mechanical properties.

The lowest MOE value of 0.39 GPa was found in L1P0 (banana stem board without surface layers). This is related to the high content of extractives in banana stems, which can degrade the bond strength between adhesive and non-wood lignocellulosic materials, thereby reducing the flexural strength of SPb. This finding shows that board modification by providing surface layers can increase the mechanical value. Specifically, banana stems with surface layers had higher MOE values, which were 8 times greater than those of the untreated boards. Iswanto et al. [9] reported that using bamboo woven and meranti veneer as surface layers on bagasse particleboards could increase the flexural strength values of the particleboard. Moreover, in this study, the MOE of SPb with coconut husk materials was generally lower than that of the other materials because of the lower density and higher springback of SPb from coconut husk.

#### 3.11.2. Bending Strength (MOR)

**Figure 19:**
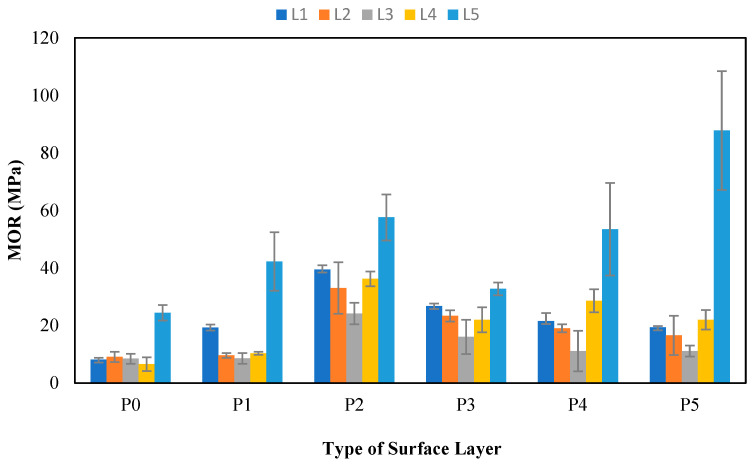
MORs of SPb fabricated from non-wood lignocellulosic materials and various surface layers. P0: without surface layers, P1: 1 mm bamboo strand, P2: 3 mm bamboo strand, P3: 1 mm wood strand, P4: 3 mm wood strand, P5: veneer, L1: banana stem, L2: rice straw, L3: coconut husk, L4: bagasse, L5: snakefruit palm fronds FVB.

The bending strength value, commonly referred to as the MOR, is a mechanical property of boards that indicates their ability to resist loads. The MOR results for the tested SPb are presented in Figure 19, which shows that the values obtained in this research ranged from 6.52 to 87.79 MPa. On the basis of the results obtained, it can be concluded that all MOR values of SPb met the JIS A 5908:2003 standard [6], which requires a value greater than 8 N/mm^2^ (8 MPa). However, the SPb samples did not meet the standard, including bagasse and banana stems without surface layers and coconut husk boards with 1 mm bamboo. This research revealed a significant relationship pattern, showing that overlaying particleboard can increase the bending strength. The trend of the MOR values follows a similar pattern to the findings of previous studies by Iswanto et al. and Subiyanto et al. [9,72].

Table 21 shows that non-wood lignocellulosic materials, surface layers, and the interaction between the two treatments significantly affected the MOR values of SPb. The highest MOR value of 87.79 MPa was found in L5P5 (snakefruit palm frond FVB particleboard with veneer surface layers). This was attributed to the absence of gaps or partitions in the surfaces of the particleboards overlaid with veneers. Similarly, Iswanto et al. [9] reported that the lowest MOR values obtained from particleboard with the shortest particle length led to more spaces between the arranged particles and weaker flexural strength. The lowest MOR value of 6.52 MPa was found in bagasse without surface layers. When correlated with the hemicellulose content of the tested nonwood lignocellulosic materials, bagasse had the highest value. However, the low MOR value was attributed to the high hemicellulose content, a weak compound with a lower degree of polymerization, because hemicelluloses absorb more water than cellulose does, leading to poor MOR [51].

#### 3.11.3. Internal Bond Strength

**Figure 20:**
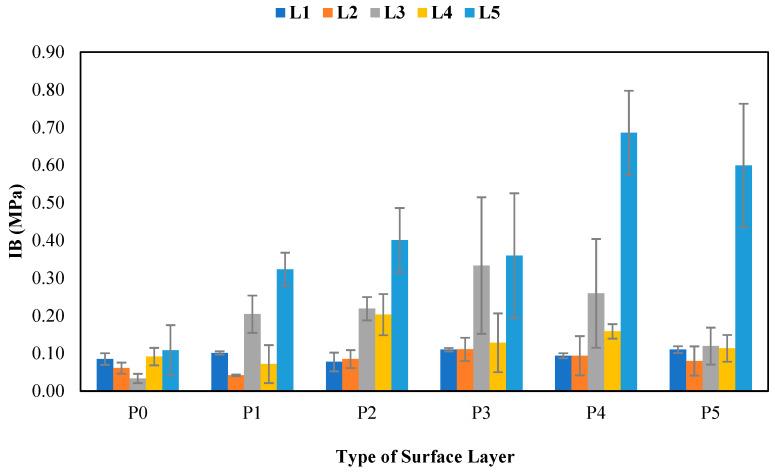
Internal bonds of SPb fabricated from non-wood lignocellulosic materials and various types of surface layers. P0: without surface layers, P1: 1 mm bamboo strand, P2: 3 mm bamboo strand, P3: 1 mm wood strand, P4: 3 mm wood strand, P5: veneer, L1: banana stem, L2: rice straw, L3: coconut husk, L4: bagasse, L5: snakefruit palm fronds FVB.

The results of the internal bond tests are presented in Figure 20. Figure 20 shows that the internal bond values ranged from 0.03–0.69 MPa. Snakefruit palm frond FVB with surface layers met the standards. Furthermore, the contact angle of snakefruit palm frond FVB was relatively small, indicating good adhesive penetration on the surface. Coconut husk without surface layers presented the lowest internal bond strength because it had the highest contact angle. Table 22 shows that non-wood lignocellulosic materials, surface layers, and the interaction between the two treatments significantly affect the internal bonding of the particleboard.

The highest internal bond value of 0.69 MPa was found in the L5P4 treatment (FVB snakefruit palm frond board with 3 mm wood strand surface layers). The internal bond strength is related to non-wood lignocellulosic material substances and the ash content. Snakefruit palm frond FVB has a relatively low ash content and extractive substances, which minimize the disruption of adhesive bonding. The holocellulose content is the highest among non-wood lignocellulosic materials, contributing significantly to increased internal bonds [73]. Moreover, the lowest internal bond value of 0.03 MPa was found in L3P0 (coconut husk board without treatment). As shown in Figure 9, coconut husk had the highest extractive substance content among the tested non-wood lignocellulosic materials. However, the low internal bond value is attributed to hindrance during the bonding process, which disrupts the adhesive bond.

### 3.12. Acoustic Properties

**Figure 21:**
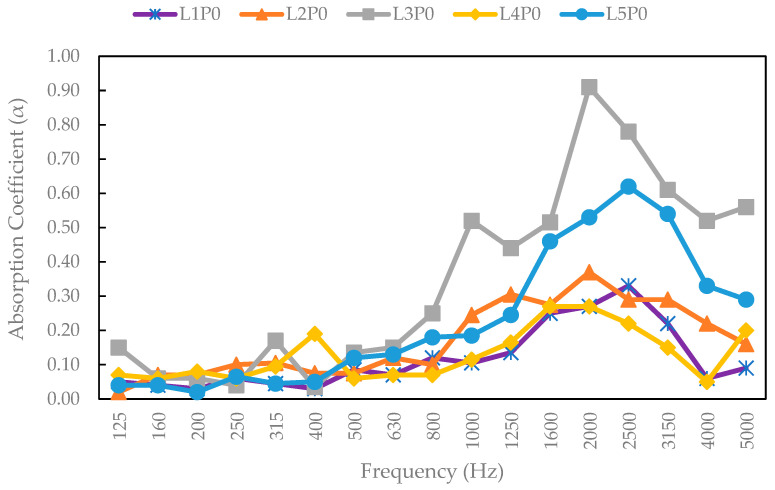
Sound absorption coefficient values for the particleboard samples fabricated from non-wood lignocellulosic materials without surface layers. L1P0 (banana stem board without a surface layer), L1P0 (rice straw board without surface layers), L3P0 (coconut husk board without surface layers), L4P0 (bagasse board without a surface layer), and L5P0 (FVB snakefruit palm frond board without surface layers).

**Figure 22:**
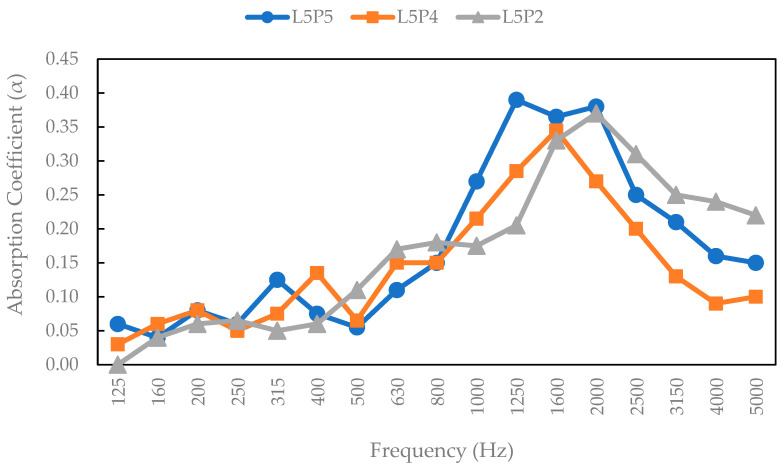
Sound absorption coefficient values for the surface layers of snakefruit palm fronds.

The acoustic properties of a few SPb samples, namely, all particleboard without overlay, were tested as representative samples, as shown in Figure 21. These included a snakefruit palm frond FVB board with 3 mm bamboo, 3 mm wood strand, and veneer surface layers. Figure 21 shows a significant variation in the sound absorption coefficient of SPb made of lignocellulosic materials without surface layers. On the basis of these results, the maximum absorption coefficient was found for coconut husk without surface layers, which provides good acoustic insulation over a fairly wide frequency range. According to Othmani et al. [74], higher porosity leads to better acoustic absorption and insulation. This is attributed to coconut husk boards without surface layers having the lowest density, which improves sound absorption. Figure 22 shows the results of the acoustic properties of SPb, indicating the absorption coefficients of snakefruit palm frond FVB particles with 3 mm bamboo strands, 3 mm wood strands, and veneer surface layers. Compared with non-wood lignocellulosic materials without adhesive, SPb had a narrower range of absorption coefficients than particleboard materials treated with surface layers. Ghofrani et al. [75] reported that particle layers affected the acoustic coefficient value, where smaller values resulted in higher reflection coefficients. Therefore, composite boards with thinner layers have higher sound absorption coefficients than those with thicker layers.

## 4. Conclusions

Analysis of the chemical components of nonwood materials such as banana stems, rice straw, coconut husks, bagasse, and snakefruit palm fronds demonstrated the potential of these materials as alternative raw materials for particleboard manufacturing. Considering the cellulose, lignin, and extractive contents of these materials, which ranged from 27 to 46%, 14 to 32%, and 4.5 to 13.5%, respectively, these materials are not inferior to wood. However, the problem with these raw materials is the low mechanical properties of the resulting board. Therefore, further efforts are needed to enhance the mechanical characteristics of boards through hybrid or sandwich particleboard techniques, such as the application of a surface coating with various materials. Notably, there was a significant increase in the strength of the board that used a surface coating. All the boards made of snakefruit palm fronds coated with various coatings presented strengths that met the minimum requirements of the JIS A 5908-2003 standard. In addition to the influence of the coating material, the higher degree of crystallinity and density of the lignocellulosic material in snakefruit palm frond compared to other materials also significantly contributed to increasing the strength of the boards.

## Figures and Tables

**Table 1:** Characteristics of the non-wood lignocellulosic materials used in this work.

Non-Wood Lignocellulosic Materials	Length (cm)	Stdev	Width (cm)	Stdev	Thickness (mm)	Stdev	Density
L1 = Banana Stem	5.13	0.14	0.54	0.12	0.63	0.41	0.16 g/cm^3^ [16]
L2 = Rice Straw	5.27	0.17	0.41	0.09	0.76	0.46	0.075 g/cm^3^ [17]
L3 = Coconut Husk	5.23	0.09	-	-	0.03	0.02	0.15 g/cm^3^ [18]
L4 = Bagasse	5.11	0.11	1.06	0.14	3.19	1.09	0.086 g/cm^3^ [19]
L5 = Snakefruit Palm Fronds	5.20	0.13	-	-	0.12	0.04	0.37–0.44 g/cm^3^ [20]

**Table 2:** Characteristics of the raw materials used for the surface layers.

Surface Layers	Length (cm)	Stdev	Width (cm)	Stdev	Thickness (mm)	Stdev
P1 = 1 mm Belangke Bamboo Strand (*Gigantochloa pruriens*)	25.21	0.17	2.91	0.32	0.92	0.25
P2 = 3 mm Belangke Bamboo Strand (*Gigantochloa pruriens*)	25.15	0.17	2.82	0.35	3.01	0.10
P3 = 1 mm Meranti Wood Strand (*Shorea* spp.)	25.14	0.15	3.00	0.18	1.01	0.13
P4 = 3 mm Meranti Wood Strand (*Shorea* spp.)	25.15	0.17	2.82	0.35	3.04	0.11
P5 = Meranti Veneer (*Shorea* spp.)	25.13	0.14	25.10	0.16	0.47	0.11

**Table 3:** Calculation of the contact angle values for the non-wood lignocellulosic materials used in this work.

No.	(L)	Repetition 1	Repetition 2	Repetition 3
1	L1	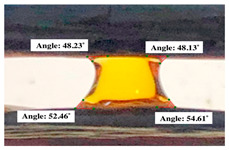	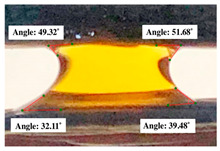	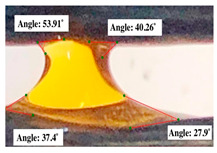
2	L2	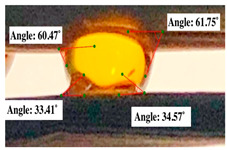	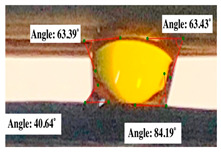	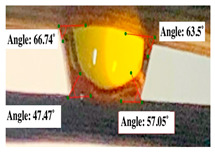
3	L3	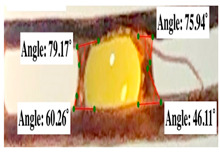	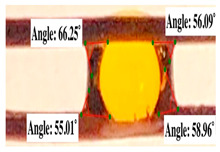	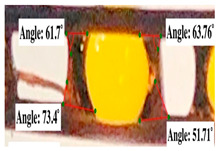
4	L4	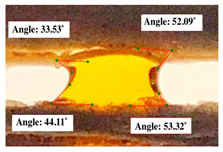	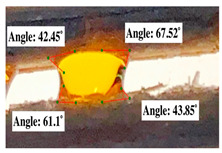	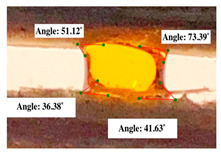
5	L5	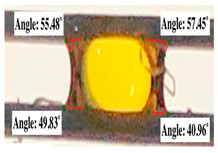	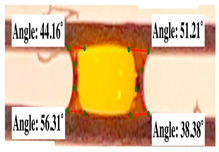	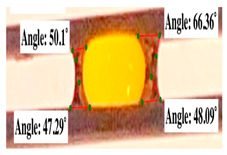

Note: L1: banana stem, L2: rice straw, L3: coconut husk, L4: bagasse, L5: snakefruit palm frond.

**Table 4:** Raw materials used to manufacture the SPb panels.

No.	Board Type	Strand Treatment	Lignocellulosic Raw Materials (g)	Adhesive (g)
1.	P0	Without Surface Layers	473.00	31.28
2.	P1	1 mm Bamboo Strand	378.49	13.90
3.	P2	3 mm Bamboo Strand	189.24	34.28
4.	P3	1 mm Wood Strand	378.49	31.57
5.	P4	3 mm Wood Strand	189.24	13.90
6.	P5	0.4 mm Veneer	435.43	31.98

**Table 5:** Standard values of the physical and mechanical property tests of SPb are produced in this work.

No.	Physical and Mechanical Properties	JIS A 5908-2003 Requirements [6]
1.	Density (g/cm^3^)	0.40–0.90
2.	Moisture Content (%)	≤14
3.	Water Absorption (%)	-
4.	Thickness Swelling (%)	≤12
5.	MOR (N/mm^2^)	≥8
6.	MOE (N/mm^2^)	≥2000
7.	Internal bond (N/mm^2^)	≥0.15

**Table 6:** *T*-test results (independent sample *t*-test) of the alpha-cellulose content.

	Coconut Husk	Banana Stem	Rice Straw	Snakefruit Palm Frond
Bagasse	0.007 *	0.012 *	0.062 ^ns^	0.013 *
Coconut Husk		0.110 ^ns^	0.018 *	0.086 ^ns^
Banana Stem			0.042 *	0.051 ^ns^
Rice Straw				0.023 *

Note: ^ns^: not significant; *: significant.

**Table 7:** *T*-test results (independent sample *t*-test) for hemicelluloses.

	Coconut Husk	Banana Stem	Rice Straw	Snakefruit Palm Frond
Bagasse	0.492 ^ns^	0.038 *	0.287 ^ns^	0.463 ^ns^
Coconut Husk		0.010 *	0.388 ^ns^	0.875 *
Banana Stem			0.034 *	0.016 *
Rice Straw				0.479 ^ns^

Note: ^ns^: not significant; *: significant.

**Table 8:** Results of the *t*-test (independent sample *t*-test) for holocellulose.

	Coconut Husk	Banana Stem	Rice Straw	Snakefruit Palm Frond
Bagasse	0.074 ^ns^	0.864 ^ns^	0.760 ^ns^	0.059 ^ns^
Coconut Husk		0.002 *	0.053 ^ns^	0.176 ^ns^
Banana Stem			0.481 ^ns^	0.030 *
Rice Straw				0.054 ^ns^

Note: ^ns^: not significant; *: significant.

**Table 9:** Results of the *t*-test (independent sample *t*-test) for lignin.

	Coconut Husk	Banana Stem	Rice Straw	Snakefruit Palm Frond
Bagasse	0.000 *	0.005 *	0.003 *	0.001 *
Coconut Husk		0.001 *	0.001 *	0.002 *
Banana Stem			0.084 ^ns^	0.002 *
Rice Straw				0.003 *

Note: ^ns^: not significant; *: significant.

**Table 10:** Results of the *t*-test (independent sample *t*-test) for extractive substances.

	Coconut Husk	Banana Stem	Rice Straw	Snakefruit Palm Frond
Bagasse	0.043 *	0.984 ^ns^	0.040 *	0.037 *
Coconut Husk		0.006 *	0.867 ^ns^	0.619 ^ns^
Banana Stem			0.002 *	0.004 *
Rice Straw				0.370 ^ns^

Note: ^ns^: not significant; *: significant.

**Table 11:** Results of the *t*-test (independent sample *t*-test) for ash content.

	Coconut Husk	Banana Stem	Rice Straw	Snakefruit Palm Frond
Bagasse	0.064 ^ns^	0.000 *	0.000 *	0.002 *
Coconut Husk		0.001 *	0.001 *	0.989 ^ns^
Banana Stem			0.004 *	0.000 *
Rice Straw				0.000 *

Note: ^ns^: not significant; *: significant.

**Table 12:** Percentage of crystallinity of the non-wood lignocellulosic materials.

Non-Wood Lignocellulosic Materials	F Crystalline (kcps deg)	F Amorphous (kcps deg)	Crystallinity (%)
L1	10.88	29.2	27.15
L2	13.15	49.09	21.13
L3	13.63	37.39	26.71
L4	12.19	38.26	24.15
L5	36.11	66.07	35.34

Description: L1: banana stem, L2: rice straw, L3: coconut husk, L4: bagasse, L5: snakefruit palm frond.

**Table 13:** Fiber dimensions of the non-wood lignocellulosic materials used in this work.

Non-Wood Lignocellulosic Materials	Fiber Dimensions							
	Length (μm)		Diameter (μm)		Lumen (μm)		Wall Thickness (μm)	
	$\bar{x}$	St.Dev	$\bar{x}$	St.Dev	$\bar{x}$	St.Dev	$\bar{x}$	St.Dev
Bagasse	2618.21	400.22	24.98	3.70	15.56	3.84	4.71	0.77
Coconut Husk	1020.36	189.61	23.28	2.40	15.34	2.05	3.79	0.94
Banana Stem	4033.79	608.96	22.70	3.36	16.34	3.64	3.18	0.57
Rice Straw	2336.90	710.56	16.92	2.83	10.98	2.47	2.97	0.35
Snakefruit Palm Frond	1756.67	70.95	20.21	1.00	6.49	0.53	6.86	0.31

**Table 14:** Fiber-derived values of the non-wood lignocellulosic raw materials.

Non-Wood Lignocellulosic Materials	Fiber Dimensions									
	Felting Power		Muhlstep Ratio		Flexibility		Runkel		Rigidity Coefficient	
	$\bar{x}$	St.Dev	$\bar{x}$	St.Dev	$\bar{x}$	St.Dev	$\bar{x}$	St.Dev	$\bar{x}$	St.Dev
Bagasse	108.05	23.27	61.37	10.03	0.62	0.09	0.66	0.27	0.19	0.04
Coconut Husk	46.79	10.11	56.58	5.56	0.66	0.04	0.50	0.13	0.16	0.04
Banana Stem	181.10	43.06	48.61	9.30	0.71	0.07	0.41	0.14	0.14	0.03
Rice Straw	122.10	39.59	58.33	5.69	0.64	0.04	0.56	0.11	0.18	0.02
Snakefruit Palm Frond	87.12	6.56	89.67	0.98	0.32	0.02	2.12	0.15	0.34	0.01

**Table 15:** Springback (%) of particleboard.

Non-Wood Lignocellulosic Materials (L)	Type of Surface Layers (P)					
	P0	P1	P2	P3	P4	P5
L1	27.06 (7.56)	27.4 (5.7)	24.96 (8.1)	27.66 (2.76)	33.06 (0.81)	26.58 (4.21)
L2	30.69 (8.34)	35.16 (2.44)	35.29 (13.39)	32.77 (3.17)	22.19 (5.74)	30.61 (1.3)
L3	64.54 (14.28)	53.13 (6.04)	46.63 (3.99)	57.55 (5.61)	39.96 (7.63)	66.73 (6.76)
L4	42.53 (3.68)	34.64 (4.79)	20.18 (2.99)	30.96 (5.09)	20.59 (2.99)	42.83 (13)
L5	51.61 (3.79)	36.54 (14.6)	25.28 (4.57)	42.55 (9.76)	21.23 (6.87)	45.16 (10.24)

Description: P0: without surface layers, P1: 1 mm bamboo strand, P2: 3 mm bamboo strand, P3: 1 mm wood strand, P4: 3 mm wood strand, P5: veneer, L1: banana stem, L2: rice straw, L3: coconut husk, L4: bagasse, L5: snakefruit palm frond FVB.

**Table 16:** Density of SPb with interactions between non-wood lignocellulosic materials and surface layers.

Non-Wood Lignocellulosic Materials (L)	Type of Surface Layers (P)					
	P0	P1	P2	P3	P4	P5
L1	0.64 ^efghij^	0.67 ^defgh^	0.81 ^ab^	0.75 ^abcd^	0.55 ^jkl^	0.70 ^cdef^
L2	0.66 ^defghi^	0.65 ^efghi^	0.72 ^bcde^	0.85 ^a^	0.55 ^jkl^	0.60 ^ghijk^
L3	0.52 ^kl^	0.56 ^ijkl^	0.74 ^bcde^	0.61 ^fghijk^	0.48 ^l^	0.58 ^hijk^
L4	0.61 ^fghijk^	0.58 ^hijk^	0.78 ^abc^	0.67 ^defgh^	0.61 ^fghijk^	0.61 ^fghijk^
L5	0.53 ^kl^	0.57 ^hijkl^	0.64 ^efghij^	0.70 ^cdefg^	0.59 ^hijk^	0.60 ^fghijk^

Description: P0: without surface layers, P1: 1 mm bamboo strand, P2: 3 mm bamboo strand, P3: 1 mm wood strand, P4: 3 mm wood strand, P5: veneer, L1: banana stem, L2: rice straw, L3: coconut husk, L4: bagasse, L5: snakefruit palm frond FVB. The same letters indicate no significant difference at the 95% confidence interval.

**Table 17:** Moisture content of SPb with interactions between non-wood lignocellulosic materials and surface layers.

Non-Wood Lignocellulosic Materials (L)	Type of Surface Layers (P)					
	P0	P1	P2	P3	P4	P5
L1	13.14 ^a^	11.81 ^b^	9.42 ^efg^	10.88 ^bc^	10.59 ^bc^	10.75 ^cd^
L2	9.29 ^efgh^	7.98 ^klmno^	8.11 ^ijklmno^	7.85 ^lmno^	8.70 ^ghijklmno^	7.75 ^mno^
L3	8.99 ^fghij^	9.79 ^def^	7.61 ^no^	8.94 ^hijklmn^	8.13 ^fghijk^	8.13 ^ijklmno^
L4	11.80 ^b^	13.78 ^a^	9.15 ^fgh^	10.12 ^cde^	10.88 ^bc^	8.49 ^ghijklmn^
L5	8.75 ^fghi^	9.02 ^jklmno^	8.03 ^op^	7.46 ^op^	8.46 ^ghijklmn^	6.54 ^p^

Description: P0: without surface layers, P1: 1 mm bamboo strand, P2: 3 mm bamboo strand, P3: 1 mm wood strand, P4: 3 mm wood strand, P5: veneer, L1: banana stem, L2: rice straw, L3: coconut husk, L4: bagasse, L5: snakefruit palm fronds FVB. The same letter indicates no significant difference at the 95% confidence interval.

**Table 18:** Water absorption of SPb with interactions between non-wood lignocellulosic materials and surface layers.

Non-Wood Lignocellulosic Materials (L)	Type of Surface Layers (P)					
	P0	P1	P2	P3	P4	P5
L1	91.87 ^abc^	66.97 ^efg^	52.00 ^fghijk^	61.79 ^efghi^	64.85 ^efghi^	66.84 ^efg^
L2	90.11 ^abc^	101.62 ^ab^	67.37 ^ef^	39.02 ^jkl^	66.50 ^efg^	61.01 ^fghi^
L3	85.48 ^bcd^	68.38 ^def^	21.23 ^mn^	45.16 ^fghij^	49.56 ^ijkl^	49.56 ^ghijkl^
L4	79.14 ^cde^	103.51 ^a^	54.73 ^fghij^	65.53 ^efg^	35.28 ^klm^	78.55 ^cde^
L5	16.14 ^n^	67.59 ^ef^	57.59 ^fghi^	47.78 ^hijkl^	34.13 ^lm^	53.52 ^fghij^

Description: P0: without surface layers, P1: 1 mm bamboo strand, P2: 3 mm bamboo strand, P3: 1 mm wood strand, P4: 3 mm wood strand, P5: veneer, L1: banana stem, L2: rice straw, L3: coconut husk, L4: bagasse, L5: snakefruit palm frond FVB. The same letter indicates no significant difference at the 95% confidence interval.

**Table 19:** Thickness swelling of SPb with interactions between non-wood lignocellulosic materials and surface layers.

Non-Wood Lignocellulosic Materials (L)	Type of Surface Layers (P)					
	P0	P1	P2	P3	P4	P5
L1	35.78 ^ab^	24.23 ^de^	12.50 ^ijk^	21.43 ^ef^	5.57 ^mn^	28.62 ^cd^
L2	33.33 ^bc^	19.48 ^efgh^	6.23 ^lmn^	12.53 ^ijk^	12.37 ^ijk^	22.12 ^ef^
L3	12.36 ^ijk^	13.78 ^hij^	3.38 ^n^	3.55 ^jklmn^	3.55 ^n^	12.30 ^ijkl^
L4	22.57 ^def^	14.70 ^ghij^	5.75 ^mn^	9.77 ^jklm^	7.48 ^klmn^	18.15 ^fghi^
L5	39.91 ^a^	20.74 ^efg^	10.18 ^jklm^	10.90 ^jklm^	10.35 ^jklm^	9.08 ^jklmn^

Description: P0: without surface layers, P1: 1 mm bamboo strand, P2: 3 mm bamboo strand, P3: 1 mm wood strand, P4: 3 mm wood strand, P5: veneer, L1: banana stem, L2: rice straw, L3: coconut husk, L4: bagasse, L5: snakefruit palm frond FVB. The same letters indicate no significant difference at a confidence interval of 95%.

**Table 20:** MOE of SPb with interactions between non-wood lignocellulosic materials and surface layers.

Non-Wood Lignocellulosic Materials (L)	Type of Surface Layers (P)					
	P0	P1	P2	P3	P4	P5
L1	0.39 ^k^	2.04 ^efghij^	2.20 ^efghi^	3.38 ^de^	1.84 ^efghijk^	2.13 ^efghi^
L2	0.95 ^hijk^	2.55 ^efg^	3.31 ^de^	5.49 ^bc^	2.13 ^efghi^	2.95 ^ef^
L3	0.73 ^ijk^	0.84 ^ijk^	0.81 ^ijk^	2.49 ^efgh^	1.06 ^ghijk^	1.12 ^ghijk^
L4	0.54 ^jk^	1.74 ^fghijk^	5.19 ^bc^	4.53 ^cd^	5.82 ^abc^	2.57 ^efg^
L5	1.90 ^efghijk^	5.33 ^bc^	6.44 ^ab^	6.10 ^ab^	6.66 ^ab^	7.34 ^a^

Description: P0: without surface layers, P1: 1 mm bamboo strand, P2: 3 mm bamboo strand, P3: 1 mm wood strand, P4: 3 mm wood strand, P5: veneer, L1: banana stem, L2: rice straw, L3: coconut husk, L4: bagasse, L5: snakefruit palm frond FVB. The same letters indicate no significant difference at a confidence interval of 95%.

**Table 21:** MORs of SPb with interactions between non-wood lignocellulosic materials and surface layers.

Non-Wood Lignocellulosic Materials (L)	Type of Surface Layers (P)					
	P0	P1	P2	P3	P4	P5
L1	8.08 ^l^	19.52 ^fghijk^	39.43 ^c^	26.76 ^defg^	21.49 ^fgh^	19.31 ^hij^
L2	9.06 ^kl^	9.57 ^jkl^	33.02 ^cde^	23.33 ^efgh^	18.99 ^fghijk^	16.58 ^ghijkl^
L3	8.44 ^l^	8.54 ^l^	24.14 ^efgh^	16.00 ^fghi^	11.10 ^hijkl^	11.10 ^ijkl^
L4	6.52 ^l^	10.30 ^ijkl^	36.20 ^cd^	21.96 ^fgh^	28.58 ^def^	21.94 ^fgh^
L5	24.38 ^efgh^	42.22 ^c^	5.59 ^b^	32.73 ^cde^	53.48 ^b^	87.79 ^a^

Description: P0: without surface layers, P1: 1 mm bamboo strand, P2: 3 mm bamboo strand, P3: 1 mm wood strand, P4: 3 mm wood strand, P5: veneer, L1: banana stem, L2: rice straw, L3: coconut husk, L4: bagasse, L5: snakefruit palm frond FVB. The same letters indicate no significant difference at a confidence interval of 95%.

**Table 22:** Internal bonding of SPb with the interaction between non-wood lignocellulosic materials and surface layers.

Non-Wood Lignocellulosic Materials (L)	Type of Surface Layers (P)					
	P0	P1	P2	P3	P4	P5
L1	0.09 ^fgh^	0.10 ^fgh^	0.08 ^gh^	0.11 ^fgh^	0.09 ^fgh^	0.11 ^fgh^
L2	0.06 ^gh^	0.04 ^gh^	0.09 ^fgh^	0.11 ^fgh^	0.09 ^fgh^	0.08 ^gh^
L3	0.03 ^h^	0.20 ^def^	0.22 ^gh^	0.33 ^bc^	0.26 ^cde^	0.12 ^fgh^
L4	0.09 ^fgh^	0.07 ^gh^	0.20 ^ef^	0.13 ^fgh^	0.16 ^efg^	0.11 ^fgh^
L5	0.11 ^fgh^	0.32 ^bcd^	0.40 ^b^	0.36 ^bc^	0.69 ^a^	0.60 ^a^

Description: P0: without surface layers, P1: 1 mm bamboo strand, P2: 3 mm bamboo strand, P3: 1 mm wood strand, P4: 3 mm wood strand, P5: veneer, L1: banana stem, L2: rice straw, L3: coconut husk, L4: bagasse, L5: snakefruit palm frond FVB. The same letters indicate no significant difference at a confidence interval of 95%.

## Data Availability

The original contributions presented in this study are included in the article. Further inquiries can be directed to the corresponding author.
